# Peripheral non-viral MIDGE vector-driven delivery of β-endorphin in inflammatory pain

**DOI:** 10.1186/1744-8069-5-72

**Published:** 2009-12-14

**Authors:** Halina Machelska, Matthias Schroff, Detlef Oswald, Waltraud Binder, Nicolle Sitte, Shaaban A Mousa, Heike L Rittner, Alexander Brack, Dominika Labuz, Melanie Busch, Burghardt Wittig, Michael Schäfer, Christoph Stein

**Affiliations:** 1 Klinik für Anaesthesiologie und operative Intensivmedizin, Freie Universität Berlin, Medizinische Fakultät Charité-Campus Benjamin Franklin, Krahmerstrasse 6, D-12207 Berlin, Germany; 2 MOLOGEN AG, Fabeckstrasse 30, D-14195 Berlin, Germany; 3 Institut für Molekularbiologie und Bioinformatik, Freie Universität Berlin, Charité-Campus Benjamin Franklin, Arnimallee 22, D-14195 Berlin, Germany; 4 Current Address: Department of Pharmacology, School of Medical Sciences, University of New South Wales, Sydney 2052, Australia; 5 Current Address: Klinik und Poliklinik für Anaesthesiologie, Universität Würzburg, D-97080 Würzburg, Germany

## Abstract

**Background:**

Leukocytes infiltrating inflamed tissue produce and release opioid peptides such as β-endorphin, which activate opioid receptors on peripheral terminals of sensory nerves resulting in analgesia. Gene therapy is an attractive strategy to enhance continuous production of endogenous opioids. However, classical viral and plasmid vectors for gene delivery are hampered by immunogenicity, recombination, oncogene activation, anti-bacterial antibody production or changes in physiological gene expression. Non-viral, non-plasmid minimalistic, immunologically defined gene expression (MIDGE) vectors may overcome these problems as they carry only elements needed for gene transfer. Here, we investigated the effects of a nuclear localization sequence (NLS)-coupled MIDGE encoding the β-endorphin precursor proopiomelanocortin (POMC) on complete Freund's adjuvant-induced inflammatory pain in rats.

**Results:**

POMC-MIDGE-NLS injected into inflamed paws appeared to be taken up by leukocytes resulting in higher concentrations of β-endorphin in these cells. POMC-MIDGE-NLS treatment reversed enhanced mechanical sensitivity compared with control MIDGE-NLS. However, both effects were moderate, not always statistically significant or directly correlated with each other. Also, the anti-hyperalgesic actions could not be increased by enhancing β-endorphin secretion or by modifying POMC-MIDGE-NLS to code for multiple copies of β-endorphin.

**Conclusion:**

Although MIDGE vectors circumvent side-effects associated with classical viral and plasmid vectors, the current POMC-MIDGE-NLS did not result in reliable analgesic effectiveness in our pain model. This was possibly associated with insufficient and variable efficacy in transfection and/or β-endorphin production. Our data point at the importance of the reproducibility of gene therapy strategies for the control of chronic pain.

## Background

Exogenous opioids (e.g. morphine), and endogenous opioid peptides, such as β-endorphin (END) and enkephalins, are powerful analgesics in animals and humans [1-3]. Compared to conventional exogenous agonists, endogenous opioids have several advantages. These include reduced probabilities of receptor downregulation and of paradoxical excitatory effects due to high non-physiological exogenous agonist concentrations at the receptor [4]. Gene therapy is an attractive strategy to enhance continuous production of endogenous opioids. The most often used vectors are recombinant viruses. Several laboratories have employed herpes simplex virus (HSV) encoding preproenkephalin (PPENK) to increase enkephalin production. Transfection of the spinal cord, trigeminal or dorsal root ganglia (DRG) with such vectors resulted in the attenuation of nociceptive behaviors in animal models of acute and pathological pain [5-13]. Similar effects were found after hind paw inoculation with HSV encoding endomorphin-2 [14] or spinal application of adenoviral and adeno-associated vectors encoding END in rodents [15,16].

The immunogenicity of viral vectors, the possibility of recombination with wild-type viruses, activation of oncogenes, and their relatively small capacity for therapeutic DNA have led to the development of non-viral vectors, mostly plasmids [17]. Gene-gun application or electroporation of plasmids encoding PPENK or END precursor proopiomelanocortin (POMC) reduced experimental pain in animals [18-23]. However, plasmids may also cause undesirable effects such as the production of antibodies (Ab) against bacterial proteins, changes in gene expression caused by the antibiotic resistance markers and immune responses to CpG dinucleotide motifs [24-26].

Non-viral, non-plasmid minimalistic immunologically defined gene expression (MIDGE) vectors may overcome these problems. MIDGE vectors are linear molecules containing only a promoter, a gene of interest and an RNA-stabilizing sequence, flanked by two short hairpin oligonucleotide sequences. Important advantages of MIDGE vectors over plasmids are small size, absence of antibiotic resistance genes and the relatively low occurrence of CpG sequences [27,28]. Gene gun delivery into dermis or onto the eye lid of MIDGE encoding interleukin-12 or cytotoxic T-lymphocyte-associated antigen-4 and interleukin-4 protected cats against experimental feline immunodeficiency virus or improved corneal graft survival in mice, respectively [29,30]. To ensure the effective transport to the nucleus and transgene expression, a nuclear localization sequence (NLS) can be attached to the MIDGE. Indeed, MIDGE-NLS encoding hepatitis B antigen enhanced antiviral immunity after intramuscular injection in mice [28]. Also, intradermal administration of MIDGE-NLS encoding LACK antigen (Leishmania homolog of receptors for activated C kinase) was protective against parasitic infection in mice [31].

The rationale underlying the present studies was to combine the advantages of MIDGE vectors and the powerful analgesic properties of opioids creating MIDGE-NLS encoding POMC to enhance the production of END for the control of prolonged inflammatory pain. We have previously shown that END-producing leukocytes accumulate in inflamed tissue, where, in response to experimental stress (swimming in cold water) or to local injection of releasing agents, the cells secrete this peptide [32-40]. Released opioids bind to opioid receptors on peripheral sensory nerve terminals, resulting in local analgesia in animals and in humans [41-46]. Importantly, these events occur in peripheral tissues and, therefore, lack side effects such as nausea, respiratory depression, dependence and addiction mediated by opioid receptors in the central nervous system [2,3]. Our major objective was to enhance the production and release of END in inflamed tissue using POMC-MIDGE-NLS to provide continuous relief of inflammatory pain.

## Methods

### Animals and induction of inflammation

Male Wistar rats (200-250 g) (Charité-Universitätsmedizin Berlin, Campus Benjamin Franklin, Berlin, Germany) received 0.15 ml of complete Freund's adjuvant (CFA; Calbiochem, La Jolla, CA) into the right hindpaw under brief isoflurane anesthesia (Abbott, Wiesbaden, Germany), and were housed individually in cages in standard experimental conditions [32,34,35]. Experiments were performed according to the guidelines of the International Association for the Study of Pain [47] and were approved by the local animal care committee (Landesamt für Gesundheit und Soziales, Berlin).

### Construction and injection of MIDGE-NLS vectors

**Figure 1:**
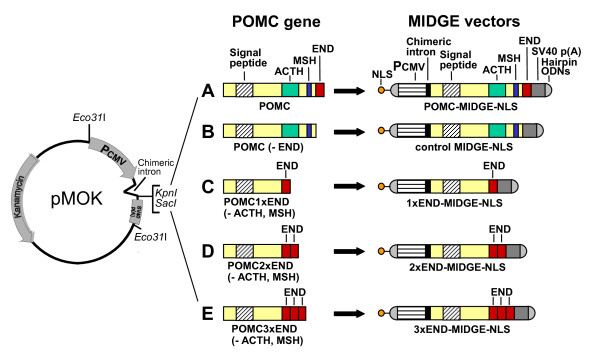
**Construction of MIDGE vectors**. **(A) **POMC exon 2-3 cDNA, encoding the signal peptide, adrenocorticotropic hormone (ACTH), melanocyte-stimulating hormone (MSH) and β-endorphin (END), was spliced into the pMOK plasmid between the SacI and KpnI restriction sites. The pMOK plasmid contains a kanamycin resistance gene, a cytomegalovirus promoter (PCMV), a chimeric intron and simian virus (SV) 40 polyadenylation (pA) site. The POMC-MIDGE-NLS was derived from the pMOK-POMC plasmid by cutting it with Eco31I, ligation with hairpin ODNs at both ends, and coupling of nuclear localization sequence (NLS) to one of the hairpin ODN. The other vectors were prepared analogously by modifying POMC gene as follows: **(B) **END sequence was removed to obtain control MIDGE-NLS; **(C) **ACTH and MSH fragments were removed while END sequence was preserved in its original place to obtain 1xEND-MIDGE-NLS encoding 1 copy of END; **(D) **ACTH fragment was removed, original END sequence was preserved and MSH fragment was replaced with an additional sequence of END to obtain 2xEND-MIDGE-NLS encoding 2 copies of END; **(E) **original END sequence was preserved and the ACTH and MSH fragments were replaced with additional END sequences to obtain 3xEND-MIDGE-NLS encoding 3 copies of END.

To prepare POMC-MIDGE-NLS the POMC exon 2-3 cDNA was amplified by RT-PCR. This is because the signal peptide essential for POMC processing along the regulated secretory pathway is encoded in exon 2 and END is encoded in exon 3 [40]. The product was cloned into the pMOK plasmid between the SacI and KpnI restriction sites and sequenced (Fig. 1A). The pMOK plasmid contains a kanamycin resistance gene, a cytomegalovirus promoter, a chimeric intron and a late SV40 pA-site (MOLOGEN AG, Berlin, Germany). The NLS peptide (PKKKRKVEDPYC; kindly provided by Dr. P. Henklein, Charité, Berlin, Germany) was coupled to 5'-GGGAGTCCAGTTTTCTGGAC in a two step procedure using a bivalent crosslinker, as previously described [28]. The resulting NLS-coupled ODN was purified by high pressure liquid chromatography. The POMC-MIDGE construct was cut out of the pMOK-POMC plasmid with Eco31I (Fermentas Life Science, Vilnius, Lithuania) and the resulting open ended expression cassette was ligated by T4 DNA ligase (Fermentas) with 5'-phosphorylated hairpin ODNs 5'-AGGGGTCCAGTTTTCTGGAC and 5'-GGGAGTCCAGTTTTCTGGAC-NLS (TIB Molbiol, Berlin, Germany). The mixture was treated with T7 DNA polymerase (Fermentas) to digest the remaining plasmid backbone. The resulting POMC-MIDGE-NLS was purified by anionic exchange column chromatography (Merck EMD-DMAE, 20 mM TRIS/HCl, 1M NaCl, pH 7).

To obtain a control vector lacking the END sequence (control MIDGE-NLS) the END sequence within the POMC coding sequence was replaced by a stop codon. The resulting product was amplified, cut with SacI and KpnI (Fermentas) and cloned into the pMOK plasmid between the SacI and KpnI restriction sites. The resulting plasmid lacking END was used to produce the corresponding control MIDGE-NLS vector (Fig. 1B), as described above.

To obtain MIDGE-NLS vectors coding for multiple copies of END the POMC expression cassette was modified by PCR as follows:

a) The adrenocorticotropic hormone (ACTH) and melanocyte-stimulating hormone (MSH) fragments were removed while END sequence was preserved in its original place. Therefore, two PCR products were generated: one consisting of the POMC sequence lacking ACTH and MSH and another one, consisting of the END sequence. The PCR products were cut with Eco31I, ligated and cloned into the pMOK plasmid after digestion with KpnI and SacI. The resulting pMOK-POMC-1xEND plasmid was used to generate the corresponding 1xEND-MIDGE-NLS vector encoding 1 copy of END (Fig. 1C), as described above.

b) The ACTH fragment was removed, the original END sequence was preserved and the MSH fragment was replaced with an additional sequence of END. Therefore, a PCR product consisting of the END sequence was amplified. The product was cut with Eco31I and SacI, ligated and cloned into the pMOK-POMC1xEND plasmid after digestion with SacI and BpiI (Fermentas). The resulting pMOK-POMC-2xEND plasmid was used to generate the corresponding 2xEND-MIDGE-NLS vector encoding 2 copies of END (Fig. 1D), as described above.

c) The original END sequence was preserved and the ACTH and MSH fragments were replaced with additional END sequences. Therefore, two PCR products consisting of the END sequence were generated. The products were cut with Eco31I and ligated. The ligated DNA fragment was cut with BpiI and SacI and cloned into the pMOK-POMC-1xEND plasmid. The resulting pMOK-POMC-3xEND plasmid was used to generate the corresponding 3xEND-MIDGE-NLS vector encoding 3 copies of END (Fig. 1E), as described above.

To examine whether MIDGE-NLS vectors can be taken up by cells in vivo we also prepared MIDGE-NLS encoding the bacterial β-galactosidase gene. The gene was amplified by PCR out of the pCMV-β plasmid (Clontech Laboratories, Mointain View, CA). Recognition sites for KpnI and XbaI (Fermentas) were generated at the ends of the product during the PCR. The product was cut with KpnI and XbaI and cloned into the pMOK plasmid between the SacI and KpnI restriction sites. The resulting pMOK-β-galactosidase plasmid was used to produce the corresponding β-galactosidase-MIDGE-NLS vector, as described above.

MIDGE vectors (25-200 μg/100 μl per paw) were injected into plantar surfaces (intraplantarly; i.pl.) of rat hindpaws at 4 days after CFA under brief isoflurane anesthesia. Further experiments were performed at 2-96 h after vector injections i.e. 4-8 days after induction of inflammation.

### Immunohistochemistry

Twenty four hours after i.pl. injections into both hindpaws of either β-galactosidase-MIDGE-NLS, POMC-MIDGE-NLS or control MIDGE-NLS (each at 50 μg) rats (n = 2) were deeply anesthetized with isoflurane and perfused transcardially. The skin with subcutaneous tissue was dissected from plantar surfaces of both hindpaws and cut into 7 μm-thick sections, which were mounted on gelatin-coated slides, as previously described [48]. Detection of β-galactosidase was performed by incubating the sections with X-gal (5-bromo-4-chloro-3-indolyl-β-D-galactopyranoside; 1 mg/ml) (Boehringer Mannheim, Vienna, Austria) as a substrate, for 4 h at 37°C [49].

To detect END the sections were incubated overnight with rabbit anti-rat polyclonal Ab against END (1:1000; Peninsula Laboratories, Merseyside, UK). Staining was performed with a vectastain avidin-biotin peroxidase complex according to the manufacturer's instructions using goat anti-rabbit biotinylated secondary Ab and avidin-biotin peroxidase (VECTASTAIN Elite Kit, Vector Laboratories, Burlingame, CA, USA), as previously described [48]. Control experiments for specificity of staining included overnight preabsorption of the primary Ab with END and omission of primary or secondary Abs.

### Flow cytometry

Twenty four hours after injections of control MIDGE-NLS (50 μg) or POMC-MIDGE-NLS (25-100 μg) into inflamed paws, rats (n = 5-6 per group) were killed with an overdose of isoflurane and plantar subcutaneous paw tissue was collected. Single cell suspensions were prepared, as described previously [35,39], and samples were stained with phycoerythrin (PE)-Cy5-conjugated mouse anti-rat CD45 (4 μg/ml; BD Biosciences, Heidelberg, Germany) to label all hematopoetic cells. To label T cells the samples were stained with mouse anti-rat CD3-PE (4 μg/ml; BD Biosciences). For intracellular stains, cells were prepared as described previously [35,39,48], and incubated with PE-conjugated mouse anti-rat RP-1 (recognizing granulocytes; 12 μg/ml; BD Biosciences) and fluorescein isothiocyanate-conjugated mouse anti-rat CD68 (formerly ED1, recognizing monocytes/macrophages; 2 μg/ml; Serotec, Oxford, UK) or 3E7, a monoclonal Ab recognizing the pan-opioid sequence Tyr-Gly-Gly-Phe at the N-terminus of opioid peptides (20 μg/ml, subtype IgG_2a_, Gramsch Laboratories, Schwabhausen, Germany), as previously described [39]. A secondary rat anti-mouse IgG_2a+b _PE Ab (0.6 μg/ml, BD Biosciences) was employed. Replacement of the primary Ab with isotype-matched nonimmune serum was used for negative controls. Absolute numbers of cells were calculated using Tru-COUNT tubes with known numbers of fluorescent beads. Data were acquired using a FACSCalibur and analyzed using the CellQuest software (all from BD Biosciences).

### Radioimmunoassay (RIA)

Twenty four hours after injections of control MIDGE-NLS (50 μg) or POMC-MIDGE-NLS (25-100 μg) into inflamed paws, rats were killed and cell suspensions from inflamed paws were prepared as for flow cytometry (see above). Cell viability as determined by Trypan blue exclusion was > 97%. Cells were pelleted and stored at -20°C. The cell pellets were lysed in RIA buffer at a concentration of 1 × 10^6 ^cells/100 μl by repetitive freeze-thaw cycles, unsoluble material was removed by centrifugation, and END immunoreactivity was measured in 100 μl of the supernatants using RIA kits (Peninsula Laboratories and Phoenix, Blomberg, Germany), as described earlier [38]. Three to seven cell samples per treatment were obtained and RIA measurements were performed in duplicates.

### Assessment of nociceptive thresholds and antinociception

Rats (n = 6-7 per group) were gently restrained and incremental pressure was applied onto the dorsal surface of the hindpaws (modified Randall-Selitto method) by means of an automated gauge (Ugo Basile, Comerio, Italy) by an experimenter blinded to the treatments. The paw pressure thresholds (PPT; cut-off at 250 g) required to elicit paw withdrawal were determined by averaging three consecutive trials separated by 10 s intervals, as previously described [32,35,48]. In the time-course experiments PPT were measured at 4 days after CFA before and at 2-96 h after vector injections. Experiments examining dose-response relationships of POMC-MIDGE-NLS and effects of vectors coding for multiple copies of END were performed at 24 h after vector administrations.

To activate endogenous opioidergic pathways of antinociception in inflamed tissue we used the cold water swim stress test. Twenty four hours after injection of MIDGE-NLS vectors the PPT were measured and animals were subjected to swimming for 1 min in a metal container filled with cold water (2-4°C). Thereafter, rats were dried and PPT were reevaluated at 1 min and 5 min after swimming, as previously described [32,35,48].

### Statistical analysis

Data are presented as means ± SEM and are expressed in raw values. Two-sample comparisons were made using the t-test for independent data and paired t-test for dependent data. Changes between several groups at one time point were analyzed by Kruskal-Wallis one-way analysis of variance (ANOVA) on ranks for not normally distributed data or by one-way ANOVA followed by the Bonferroni t-test for normally distributed data. Changes between two groups over time were evaluated by two-way ANOVA for repeated measurements followed by the Bonferroni t-test. Differences were considered significant if p < 0.05.

## Results

### POMC-MIDGE-NLS transfection efficiency in vivo

**Figure 2:**
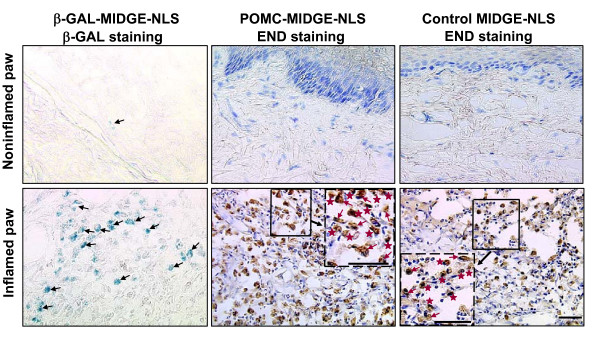
**Effects of intraplantar POMC-MIDGE-NLS on β-endorphin expression in paws**. β-galactosidase (β-GAL)-MIDGE-NLS, POMC-MIDGE-NLS or control MIDGE-NLS (each at 50 μg) were injected into both hindpaws at 4 days after induction of inflammation. Staining was performed 24 h later. Positive staining for β-GAL (in blue), particularly visible in inflamed paws, indicates that MIDGE vectors can transfect cells (some marked with black arrows) in vivo. β-endorphin (END; brown staining) is expressed in immune cells after treatment with POMC-MIDGE-NLS or control MIDGE-NLS. Morphologically these cells appear as polymorphonuclear (red arrows) and mononuclear cells (red stars), shown in the dash-boxed inserts at a higher magnification of the respective solid line boxes. Bars = 20 μm.

To examine whether MIDGE-NLS vectors can be taken up by cells in vivo rats received MIDGE-NLS encoding the bacterial β-galactosidase gene into both hindpaws (50 μg; i.pl.) 4 days after CFA. Histochemistry performed 24 h after vector injection (i.e. 5 days after CFA) revealed staining for β-galactosidase. This was visible predominantly in inflamed paws, while only weak β-galactosidase staining was seen in noninflamed paws (Fig. 2), similar to other studies [50], indicating that MIDGE vectors can transfect cells in vivo. Next, rats received bilateral i.pl. injections of either POMC-MIDGE-NLS (50 μg) or control MIDGE-NLS (50 μg). Immunohistochemical staining with END-specific Ab showed numerous immunoreactive cells in inflamed, but not in noninflamed, paws (Fig. 2). Morphologically these cells resembled polymorphonuclear and mononuclear leukocytes, as shown in our previous studies [38,48]. We did not observe END staining in any other cell types including neurons. There were no detectable differences in END-staining between POMC-MIDGE-NLS and control MIDGE-NLS (Fig. 2). Control experiments for specificity of staining did not show END-immunoreactivity (not shown).

Quantitative analysis with flow cytometry demonstrated an accumulation of granulocytes, monocytes/macrophages and T cells in inflamed paws. Using the 3E7 Ab recognizing opioid peptides (END, Met-enkephalin and dynorphin A), we found no significant differences in leukocyte subpopulations and in numbers of opioid-containing immune cells between POMC-MIDGE-NLS and control MIDGE-NLS (p > 0.05, one-way ANOVA; Table 1).

**Table 1:** Quantification of leukocyte subpopulations and of opioid-containing leukocytes in inflamed paws following intraplantar delivery of POMC-MIDGE-NLS.

Treatment	Total cell counts (× 10^3^) per paw			
	Granulocytes	Monocytes/ macrophages	T cells	Opioid cells
control MIDGE-NLS	44 ± 13	823 ± 132	62 ± 12	403 ± 65
50 μg				
POMC-MIDGE-NLS				
25 μg	56 ± 19	566 ± 89	60 ± 14	262 ± 42
50 μg	92 ± 16	633 ± 56	51 ± 6	358 ± 38
100 μg	99 ± 40	691 ± 159	50 ± 11	379 ± 91

Leukocyte counts were determined by flow cytometry. There were no significant differences between control MIDGE-NLS and POMC-MIDGE-NLS (p > 0.05, one-way ANOVA).

### Effects of POMC-MIDGE-NLS on inflammatory pain and on END content in immune cells

**Figure 3:**
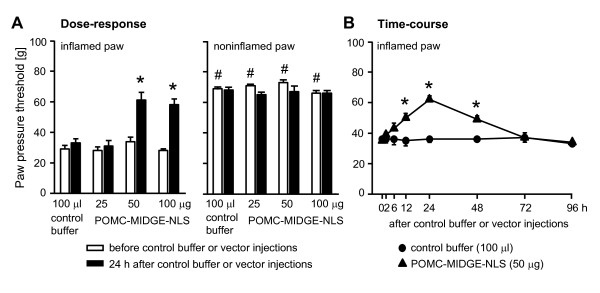
**Effects of intraplantar POMC-MIDGE-NLS on inflammatory pain**. **(A) **Paw pressure thresholds (PPT) were measured at 4 days after induction of inflammation (white bars), then control buffer (100 μl) or POMC-MIDGE-NLS (25-100 μg/100μl) were injected into inflamed paws and PPT were re-evaluated 24 h later (black bars). *p < 0.05, compared with control buffer at 24 h after its injection (one-way ANOVA, Bonferroni t-test); ^#^p < 0.001, compared with respective PPT of inflamed paws before control buffer or vector injections (depicting the development of inflammatory hyperalgesia) (paired t-test). **(B) **Time-course was evaluated at 4 days after induction of inflammation before (0 h) and at 2-96 h after control buffer or vector injections (i.e. up to 8 days following induction of inflammation). *p < 0.05, compared with control buffer (two-way repeated measures ANOVA, Bonferroni t-test).

**Figure 4:**
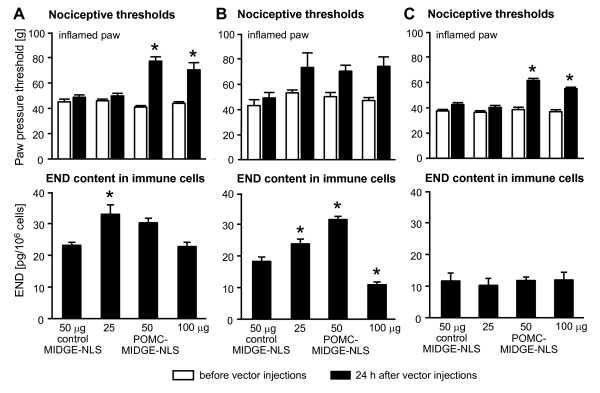
**Comparison of POMC-MIDGE-NLS effects on nociceptive thresholds and END content in immune cells in inflamed paws**. The figure shows three separate sets of experiments to test reproducibility (**A**, **B**, **C**). Control MIDGE-NLS (50 μg) and POMC-MIDGE-NLS (25-100 μg) were injected into inflamed paws at 4 days after induction of inflammation. Effects of vectors on paw pressure thresholds (PPT; upper panels) and on END content in immune cells (lower panels) were measured in parallel 24 h after their injections (black bars). PPT were also measured before vector injections (white bars). *p < 0.05, compared with control MIDGE-NLS at 24 h after its injection (one-way ANOVA, Bonferroni t-test).

**Figure 5:**
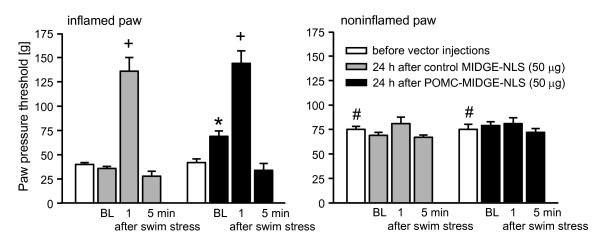
**Swim stress and POMC-MIDGE-NLS effects on inflammatory pain**. Paw pressure thresholds (PPT) were measured at 4 days after induction of inflammation (white bars), then control MIDGE-NLS (50 μg; gray bars) or POMC-MIDGE-NLS (50 μg; black bars) were injected into inflamed paws and PPT were re-evaluated 24 h later (baseline, BL). Next, rats were exposed to cold water swim stress and PPT were re-examined 1 min and 5 min later. *p < 0.05, compared with the BL of control MIDGE-NLS; ^+^p < 0.05, compared with respective BL (two-way repeated measures ANOVA and Bonferroni t-test); ^#^p < 0.001, compared with respective PPT of inflamed paws before vector injections (depicting the development of inflammatory hyperalgesia) (paired t-test).

**Figure 6:**
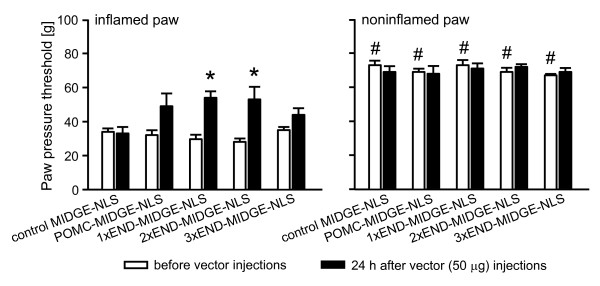
**Effects of intraplantar POMC-MIDGE-NLS encoding multiple copies of β-endorphin (END) on inflammatory pain**. Paw pressure thresholds (PPT) were measured at 4 days after induction of inflammation (white bars), control MIDGE-NLS, POMC-MIDGE-NLS and vectors with 1-3 copies of END (1-3xEND-MIDGE-NLS) (each at 50 μg) were injected into both hind paws and PPT were re-evaluated 24 h later (black bars). *p < 0.05, compared with control MIDGE-NLS at 24 h after its injection (t-test); ^#^p < 0.001, compared with respective PPT of inflamed paws before vector injections (depicting the development of inflammatory hyperalgesia) (paired t-test).

Four days after induction of inflammation rats developed mechanical hyperalgesia manifested by decreased PPT in inflamed compared with contralateral noninflamed paws, before control buffer or vector injections (p < 0.001, Fig. 3, Fig. 4A; p < 0.01, Fig. 4B; p < 0.001, Fig. 4C, Fig. 5, Fig. 6) (paired t-test). In Fig. 3B and Fig. 4, for clarity of figures, only PPT in inflamed paws are shown. We have previously shown that PPT in contralateral paws are comparable to PPT before induction of inflammation [51]. There were no significant differences in PPT of inflamed paws among groups within each experimental set (p > 0.05, one-way ANOVA; Fig. 3A, Fig. 4A, B, C, Fig. 5, Fig. 6). To examine a possible variability in the degree of hyperalgesia between different sets of experiments (i.e. different figures), we compared basal PPT in inflamed paws of control groups (as representative groups) in all figures with each other. Statistical analysis revealed that PPT in Fig. 4A and 4B were slightly but significantly higher compared with PPT in Fig. 3A (p < 0.05, one-way ANOVA, Bonferroni t-test). Equivalent analysis of PPT in noninflamed paws revealed no significant differences (p > 0.05, one-way ANOVA; Fig. 3, Fig. 4, Fig. 5, Fig. 6).

Injections of control phosphate buffer (100 μl) into inflamed paws did not significantly change PPT in inflamed paws or in contralateral noninflamed paws (p > 0.05, paired t-test; Fig. 3A). At 24 h after injection into inflamed paws, POMC-MIDGE-NLS (50-100 μg) produced PPT elevations in inflamed (p < 0.05, one-way ANOVA, Bonferroni t-test) but not in contralateral noninflamed paws (p > 0.05, one-way ANOVA) (Fig. 3A). POMC-MIDGE-NLS (50-100 μg) reversed mechanical hyperalgesia reaching the thresholds of noninflamed paws (Fig. 3A). Higher doses of POMC-MIDGE-NLS (200 μg) did not further increase the PPT in inflamed (58 ± 3.4 g vs. 56 ± 12 g, 100 μg vs. 200 μg) or in noninflamed paws (66 ± 1.7 g vs. 64 ± 1.5 g, 100 μg vs. 200 μg) (p > 0.05, t-test). POMC-MIDGE-NLS (50 μg) injected into inflamed paws produced significant elevations of PPT for 48 h in these paws (p < 0.05, two-way repeated measures ANOVA, Bonferroni t-test; Fig. 3B). There were no significant changes in the PPT of inflamed paws in the group receiving control buffer (Fig. 3B) or in the contralateral noninflamed paws (p > 0.05, two-way repeated measures ANOVA; not shown).

Next, we examined whether there is a correlation between PPT elevations and END content in immune cells harvested from inflamed paws at 24 h following i.pl. MIDGE-NLS vector injections. We performed three separate sets of experiments to test reproducibility. In one set, POMC-MIDGE-NLS elevated PPT of inflamed paws significantly at 50 μg and 100 μg (p < 0.05, one-way ANOVA, Bonferroni t-test; Fig. 4A), similar to Fig. 3A. POMC-MIDGE-NLS (25 μg and 50 μg) also enhanced END content in leukocytes, however, a significant effect was observed only after 25 μg (p < 0.05, one-way ANOVA, Bonferroni t-test; Fig. 4A). In another experiment, POMC-MIDGE-NLS (25-100 μg) slightly but non-significantly increased PPT (p > 0.05, one-way ANOVA; Fig. 4B). Leukocytic END content was significantly elevated at 25 μg and 50 μg, and decreased at 100 μg (p < 0.05, one-way ANOVA, Bonferroni t-test; Fig. 4B). In the third set of experiments, POMC-MIDGE-NLS (50-100 μg) significantly elevated PPT (p < 0.05, one-way ANOVA, Bonferroni t-test; Fig. 4C) but did not produce significant changes in the END content (p > 0.05, one-way ANOVA on ranks; Fig. 4C). The control levels of END in Fig. 4A and Fig. 4B were higher than those in Fig. 4C, although a significant difference was found only between Fig. 4A and Fig. 4C (p < 0.05) but not between Fig. 4B and Fig. 4C (p > 0.05) (one-way ANOVA, Bonferroni t-test). Together, in all three experiments POMC-MIDGE-NLS elevated nociceptive thresholds in inflamed paws to a similar degree, but the effects were moderate and not always reached statistical significance. POMC-MIDGE-NLS modestly enhanced END content in immune cells in some experiments. However these effects were not always correlated with the effects on nociceptive thresholds.

### Swim stress and POMC-MIDGE-NLS effects on inflammatory pain

We have previously shown that exposure of rats to a cold water swim stress leads to the local release of opioid peptides from immune cells resulting in antinociception restricted to the inflamed paws [32,34-36,48]. This stress paradigm does not significantly change nociceptive thresholds in animals without inflammation [52]. Here, we examined whether swim stress can stimulate the secretion of END, possibly overcoming the variability in its leukocytic content, and increase its anti-hyperalgesic actions. The control vector did not significantly change PPT in inflamed paws or in contralateral noninflamed paws (p > 0.05, paired t- test; Fig. 5). In this experiment, injection of POMC-MIDGE-NLS (50 μg) into inflamed paws significantly elevated the PPT compared with control MIDGE-NLS (50 μg) 24 h later in inflamed, but not in contralateral noninflamed, paws (p < 0.05 and p > 0.05, respectively; Fig. 5; compare baselines of control MIDGE-NLS and POMC-MIDGE-NLS). Subsequently, rats were exposed to swim stress. In both control MIDGE-NLS- and POMC-MIDGE-NLS-treated groups there were significant elevations of PPT at 1 min after swim stress in inflamed paws (p < 0.05), but there were no significant differences between the treatments (p > 0.05) (Fig. 5; all comparisons were analyzed with two-way repeated measures ANOVA and Bonferroni t-test). There were also no significant changes in contralateral noninflamed paws (p > 0.05, two-way repeated measures ANOVA; Fig. 5). Thus, swim stress produced similar antinociceptive actions after POMC-MIDGE-NLS as compared with control MIDGE-NLS.

### Effects of POMC-MIDGE-NLS encoding multiple copies of END on inflammatory pain

All vectors were injected into both hind paws in a dose of 50 μg, at 4 days after CFA and PPT were measured 24 h later. Control MIDGE-NLS did not significantly change PPT in inflamed or in contralateral noninflamed paws (p > 0.05, paired t-test; Fig. 6). 1xEND-MIDGE-NLS and 2xEND-MIDGE-NLS significantly, but slightly, elevated PPT compared with the control vector (p < 0.05; t-test; Fig. 6). POMC-MIDGE-NLS and 3xEND-MIDGE-NLS did not significantly change PPT compared with the control vector (p > 0.05, t-test; Fig. 6). The PPT in contralateral noninflamed paws were not significantly changed by any of the treatments (p > 0.05, t-test; Fig. 6).

## Discussion

In the present study we examined the control of prolonged inflammatory pain with novel END-coding MIDGE vectors, which lack the disadvantages of classical viral and plasmid vectors. Although, POMC-MIDGE-NLS injected into inflamed tissue appeared to be taken up by leukocytes in vivo (Fig. 2, Fig. 4), its anti-hyperalgesic actions were rather moderate and not consistently reproducible (Fig. 3, Fig. 4, Fig. 5, Fig. 6). Also, these effects could not be enhanced by increasing END release through stress or by modifying POMC-MIDGE-NLS to code for multiple copies of END (Fig. 5, Fig. 6).

Previously we have consistently observed significant analgesic effects of both exogenous and leukocyte-derived opioids against mechanical stimulation in experimental and clinical inflammatory pain [2,3]. In the current study, i.pl. injection of POMC-MIDGE-NLS reversed mechanical hyperalgesia, i.e. nociceptive thresholds returned to the levels measured in contralateral noninflamed paws in some experiments (Fig. 3, Fig. 4A, Fig. 5). Nevertheless, the effects were rather moderate and not always reproducible (Fig. 4B, C, Fig. 6). This lack of consistently significant anti-hyperalgesic effects of POMC-MIDGE-NLS might be related to higher basal PPT (after CFA but before vector injections) and bigger within-group variability of responses in some experiments (Fig. 4B, Fig. 6). In some previous reports hyperalgesia was fully reversed by opioid peptide vector delivery. For example, intrathecal electroporation of modified POMC plasmids suppressed thermal hyperalgesia in a neuropathic pain model [23], and PPENK-HSV applied to the paw skin reversed thermal hyperalgesia induced by pertussis toxin or capsaicin [5,11,12]. However, similar to our results, the majority of studies using either HSV, adenovirus, adeno-associated or plasmid vectors encoding opioid peptides found only partial and modest reductions of thermal or mechanical hyperalgesia in long-lasting (weeks) or short-lasting (1-3 h) pain models [8-10,13,15,16,18-23]. A reproducibility of anti-hyperalgesic effects has not been reported in those earlier studies.

POMC-MIDGE-NLS decreased hyperalgesia for about 2 days. This is much shorter than the effects measured for 2-20 weeks after injections of viral vectors encoding opioid peptides [5,7-14,16], or up to 2 weeks after endomorphin-2-HSV or POMC plasmid applications [14,20,23]. However, in other studies using END-coding adenoviral vector or POMC plasmids, attenuation of hyperalgesia was only measured in short-lasting (30 min-3 h) pain models [15,18,19,21,22]. Thus, longer-lasting anti-hyperalgesic effects were mostly produced by viral vectors preferentially targeting neurons [5,7-14,16]. In contrast, in our model MIDGE-NLS appeared to be taken up mostly by immune cells. At this stage of inflammation (5 days following CFA) monocytes/macrophages dominate, and their turnover rate of several hours correlates with the time-course of POMC-MIDGE-NLS-mediated anti-hyperalgesia. On the other hand, the 48 h-lasting elevation of nociceptive thresholds produced by POMC-MIDGE-NLS is superior to the 5-10 min-lasting effects after i.pl. END injection [48]. Hence, some of our experiments demonstrated a clear prolongation of anti-hyperalgesic actions by POMC-MIDGE-NLS-based END delivery. Modification of POMC-MIDGE-NLS vectors to also transfect neurons might be an interesting future approach for improvement of their anti-hyperalgesic effects.

In some of our experiments POMC-MIDGE-NLS slightly elevated END content in immune cells accumulating in inflamed tissue. However, these effects were not always statistically significant or directly correlated with the attenuation of hyperalgesia, indicating variability in transfection and/or END production efficacy. Although there was some variability in the control END levels among experimental sets (Fig. 4) this does not seem to predict the efficacy of POMC-MIDGE-NLS in END production. For example, even though the control END levels in Fig. 4A and Fig. 4B were comparable, the END levels after different doses of POMC-MIDGE-NLS varied between the two graphs. Also, it seems that there was no direct correlation between the control END levels and the extent of hyperalgesia. Thus, despite differences in the control END levels in Fig. 4A, B and 4C, the basal PPT (after CFA but before control vector injection) were comparable among these three graphs. The lack of significant increases in the numbers of opioid-positive leukocytes suggests that when POMC-MIDGE-NLS transfected these cells it was mostly active in those already expressing the peptide. Other vectors have been shown to increase opioid peptide production in different tissues. For example, HSV-PPENK applied to skin enhanced the expression of PPENK, Met- or Leu-enkephalin in the spinal cord and primary afferent neurons in healthy animals [5,7,11-13]. Spinal application of adenoviral vectors encoding END resulted in increased cerebrospinal fluid levels of the peptide [15]. Importantly, while antinociceptive effects were assessed after induction of inflammation or neuropathy, the transfection efficacy of these viral vectors in peripheral neurons was primarily verified in healthy animals [5,7,11-13,16]. There was no clear explanation for these approaches and they make interpretation of the data difficult. Transfection efficacy of plasmid vectors was evaluated either directly in inflamed tissues treated with the vectors, or in the spinal cord after intrathecal electroporation. For instance, gene-gun application of plasmids coding for POMC or PPENK to inflamed rat paws or into the bladder wall, or intrathecal electroporation in animals with hind paw inflammation or with sciatic nerve injury increased levels of END or Met-enkephalin in the respective tissues, although cell types were not specified in those experiments [18-23]. None of those previous studies reported transfection of immune cells with vectors encoding opioid peptide precursors. MIDGE vectors do not seem to possess particular characteristics in cell type targeting. In previous studies their transfection efficacy was examined predominantly in vitro in various cell lines. Accordingly, MIDGE-NLS encoding hepatitis B antigen transfected chicken hepatoma cells [28], MIDGE-NLS encoding LACK antigen was successful in transfecting kidney COS-7 cells [31], and MIDGE encoding IL-12 transfected a feline T lymphocyte line [29]. Hence, it appears that if a vector is introduced into a cell line or tissue, cells are "forced" to express a transgene regardless of the physiological/pathological state of the tissue. This might explain some (scarce) staining in non-inflamed paws after β-galactosidase-MIDGE-NLS application in our current study. On the other hand, it is reasonable to assume that the type of transfected cells might be determined by the pathological condition of the tissue. Thus, in our studies it appears that when tissue is inflamed and infiltrated by leukocytes, these cells might be a major target for POMC-MIDGE-NLS.

Our studies do not provide direct evidence for the opioid-receptor selectivity and the site of anti-hyperalgesic actions of END coded by POMC-MIDGE-NLS. Testing specific and peripherally-restricted opioid receptor antagonists would be necessary to determine selectivity and site of the vector actions. However, because of the variable reproducibility of POMC-MIDGE-NLS-mediated anti-hyperalgesic effects, such experiments are difficult to perform. Similar concerns apply to the effects of POMC-MIDGE-NLS encoding 1 or 2 copies of END, as they produced only minor PPT elevations. Nevertheless, an action via peripheral opioid receptors is most likely. The fact that Abs recognizing endogenous END were reactive in our immunohistochemical and RIA experiments suggests that POMC-MIDGE-NLS-induced END is not different from authentic END and therefore should act at opioid receptors. We have previously shown that opioid peptides injected directly into inflamed tissue can produce antinociception via opioid receptors on peripheral sensory neurons [2,3,48]. Furthermore, direct application to injured tissues of either HSV-PPENK or POMC plasmid resulted in peripheral opioid receptor-selective anti-hyperalgesic effects [6,7,13,21]. In contrast, applications of POMC-MIDGE-NLS (Fig. 6), POMC plasmid or of exogenous opioids into peripheral non-injured tissues [2,3,21,48] did not significantly change nociceptive thresholds. Most probably this is related to an intact perineural barrier and/or insufficient number or G-protein coupling of opioid receptors on sensory neurons in noninflamed tissues [2,3,43,44,46].

## Conclusion

In this study we have evaluated the effectiveness of a novel non-viral, non-plasmid MIDGE-NLS vector coding for END in the control of prolonged inflammatory pain. We show evidence of an enhanced antinociceptive response, uptake by immune cells and increased content of END. However, these effects were not always reproducible or directly correlated with each other. Thus, while MIDGE based vectors were effective against infections and in improving graft survival [28-31] anti-hyperalgesic effects of POMC-MIDGE-NLS were not reliably reproducible in our studies. Enhancing opioid secretion from leukocytes by stress [32,34,35,48] did not improve these effects, although this might have been masked by a relatively strong swim-stress induced antinociception. Further, the modification of POMC-MIDGE-NLS vectors to code for multiple copies of END did not produce better effects either. Thus, variability in transfection and/or END production efficiency of POMC-MIDGE-NLS appears to limit their potential antinociceptive actions in our model of painful paw inflammation. Noteworthy, our studies show that pain modulation by gene therapy might be susceptible for vector transfection variability, which should be addressed in future studies.

## Competing interests

The authors declare that they have no competing interests.

## Authors' contributions

HM designed and conducted most behavioral experiments, analyzed the data and wrote the manuscript. M. Schroff, DO and BW designed and constructed the MIDGE vectors. WB and DL performed some behavioral experiments. NS and MB executed radioimmunoassay. SAM carried out immunohistochemistry. HLR and AB commenced flow cytometry experiments. M. Schäfer participated in the study design. CS and BW conceived the study. CS participated in the data interpretation and writing of the manuscript. All authors have read and approved the final manuscript.
